# Flavanones as Modulators of Gut Microbiota and Cognitive Function

**DOI:** 10.3390/molecules30102203

**Published:** 2025-05-18

**Authors:** Natalia Cichon, Rafał Szelenberger, Maksymilian Stela, Marcin Podogrocki, Leslaw Gorniak, Michal Bijak

**Affiliations:** Biohazard Prevention Centre, Faculty of Biology and Environmental Protection, University of Lodz, Pomorska 141/143, 90-236 Lodz, Poland; natalia.cichon@biol.uni.lodz.pl (N.C.); rafal.szelenberger@biol.uni.lodz.pl (R.S.); maksymilian.stela@biol.uni.lodz.pl (M.S.); marcin.podogrocki@biol.uni.lodz.pl (M.P.); leslaw.gorniak@biol.uni.lodz.pl (L.G.)

**Keywords:** flavanones, naringenin, hesperidin, gut–brain axis, microbiota, cognitive health

## Abstract

Flavanones, a key subclass of flavonoids, exhibit a wide range of biological activities, including antioxidant, anti-inflammatory, and neuroprotective properties. Predominantly found in citrus fruits, they occur in both aglycone and glycosylated forms, undergoing extensive metabolic transformation upon ingestion. Recent evidence suggests that flavanones, such as naringenin and hesperidin, influence gut microbiota composition, fostering a balance between beneficial and pathogenic bacterial populations. The gut microbiota plays a pivotal role in regulating the gut–brain axis, impacting cognitive function through the production of short-chain fatty acids (SCFAs), neurotransmitters, and anti-inflammatory cytokines. The modulation of the gut microbiome by flavanones has been associated with improvements in cognitive performance and a reduced risk of neurodegenerative disorders. This review provides a comprehensive analysis of the characteristics of major flavanones, their metabolic pathways, and their impact on gut microbiota and cognitive function. It covers the fundamental mechanisms through which flavanones exert their effects, as well as their potential therapeutic applications for brain health and neuroprotection. Despite promising findings, further research is needed to determine optimal dosages, strategies to enhance bioavailability, and long-term safety profiles.

## 1. Introduction

Flavonoids are a class of naturally occurring polyphenolic compounds ubiquitously present in plants. As a secondary metabolite, flavonoids are produced through the phenylpropanoid pathway and possess a variety of health benefits, including anti-inflammatory, antioxidant, anticancer, neuroprotective, antimicrobial, and antiviral features [1,2].

Flavonoids typically share a common structure known as the flavan nucleus, which consists of two aromatic rings (A and B) connected by a pyran ring (C). The position of the B-ring’s linkage to the C-ring differentiates flavonoids (2-phenylbenzopyrans), isoflavonoids (3-phenylbenzopyrans), and neoflavonoids (4-phenylbenzopyrans). Among these, the most prevalent group is the 2-phenylbenzopyrans, which can be further categorized into 3-hydroxyflavonoids (including flavonols, flavanols, anthocyanidins, and dihydroflavonols), and flavonoids lacking a substituent at the C3 position (such as flavanones and flavones) [3].

Flavanones, also known as dihydroflavones, represent a subclass of flavonoids, predominantly found in citrus fruits, including lemons, limes, oranges, grapefruits, mandarins, clementines, tangelos, and grapes [4,5]. A key structural difference between flavanones and other flavonoid compounds is a saturated C-ring and the absence of a double bond between positions C2 and C3 in the C-ring. Moreover, their structure includes hydroxyl groups at positions C5 and C7 of the A-ring, as well as hydroxyl or methoxy substituents at positions C3 or C4 of the B-ring [4].

Flavanones exist in two primary forms, glycosides and aglycones, which differ structurally, chemically, and functionally. These differences are crucial for understanding their behavior in plants, their metabolism in humans, and their biological activity. Primarily, flavanones occur in citrus as glycosylated derivatives, where a sugar moiety is attached to the aglycone (flavanone core without any sugar moieties) via an O-glycosidic bond, mainly at the C7 position of the A-ring. Common sugar moieties include glucose, rhamnose, or complex disaccharides such as rutinose or neohesperidose moieties, which are L-rhamnosyl-D-glucosyl derivatives differing in their interglycosidic linkages (α-1,6 for rutinose and α-1,2 for neohesperidose) [6].

The primary diet source of flavanones for humans is the consumption of fresh fruits or squeezed juices. The content of individual flavanones varies among different fruits. In blonde or blood sweet oranges, the highest concentration was observed for hesperidin (200–600 mg/L). Moreover, hesperidin was also the predominant flavanone in limes and lemons (38–410 mg/L), as well as in juice obtained from *C. clementina* (50–850 mg/L). In grapefruits, the highest concentration was found for naringin (48–1220 mg/L), whereas in tangelos, neohesperidin was the most abundant (100–400 mg/L) [6].

**Figure 1:**
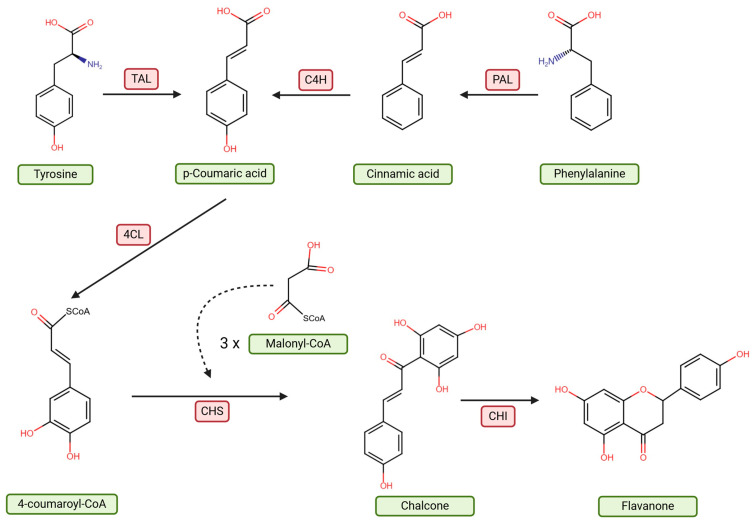
Schematic representation of flavanones’ biosynthesis. Abbreviation: 4CL—4-coumaryl: CoA ligase; C4H—Cinnamate-4-hydroxylase; CHI—chalcone isomerase; CHS—type III polyketide synthase chalcone synthase; PAL—phenylalanine ammonia-lyase; TAL—tyrosine ammonia-lyase. Created in BioRender. Bijak, M. (2025) https://BioRender.com/n94b151, accessed on 15 May 2025, [6,7,8].

Flavonones are synthesized from two amino acids (Figure 1): tyrosine and phenylalanine. The transformation process begins with two enzymes: phenylalanine ammonia-lyase (PAL), which removes an amino group from L-phenylalanine to form trans-cinnamic acid; and tyrosine ammonia-lyase (TAL), which deaminates L-tyrosine, thereby removing an amino group and directly converting it into p-coumaric acid. Cinnamate-4-hydroxylase (C4H) introduces a 4′-hydroxyl group to the phenyl ring in trans-cinnamic acid, also forming p-coumaric acid. Further, 4-coumaryl: CoA ligase (4CL) attaches the coenzyme A (CoA) to the carboxyl group, resulting in the formation of 4-coumaroyl-CoA. In the next step, the type III polyketide synthase chalcone synthase (CHS) catalyzes the stepwise condensation of three malonyl-CoA with one 4-coumaroyl-CoA, forming chalcones. The final step in the biosynthesis of flavanones involves the isomerization of chalcones. Chalcone isomerase (CHI) catalyzes the conversion of chalcones into their corresponding flavanones via the rearrangement of the chalcone structure, specifically resulting in the intramolecular cyclization of chalcones into flavanones [6,7,8].

Due to their bioactive properties, flavanones have attracted considerable attention for their potential health benefits. To fully understand their therapeutic mechanisms, it is essential to examine their chemical characteristics and the complex metabolic processes they undergo in the body. The purpose of this review is to explore the potential role of flavanones in modulating gut microbiota and their consequent effects on cognitive function, with a focus on underlying mechanisms and therapeutic implications.

## 2. Characteristics of Flavanones and Their Metabolism in the Body

### 2.1. Naringenin

Naringenin (5,7-Dihydroxy-2-(4-hydroxyphenyl)chroman-4-one) is a flavanone aglycone derived from naringin, commonly present in both its free and glycosylated forms in various plant species [9]. Its primary dietary sources include citrus fruits, particularly *Citrus aurantium* var. *sinensis* (oranges), *Citrus reticulata Blanco* (tangerines), and *Citrus maxima* (pomelo). However, the highest concentrations of this flavonoid are found in *Citrus paradisi Macfad*. (grapefruit) and *Citrus aurantium* var. *amara* (bitter oranges). Additional natural sources include *Pistacia vera* (pistachios), *Prunus dulcis* (almonds), *Tamarindus indica* (Indian tamarind seeds), and *Benincasa hispida* (winter melon). Naringenin is also found in *Cyclopia intermedia* (honeybush tea), propolis, and the sprouts of certain plants, such as *Brassica oleracea* var. *italica* (broccoli) and *Lens culinaris* (lentils). In tomatoes (*Solanum lycopersicum*), naringenin chalcone is present and is converted into free naringenin during food processing [10].

The chemical structure of naringenin consists of a flavanone skeleton with hydroxyl groups at positions 5′ and 7′ on ring A and at position 4′ on ring B, contributing to its strong antioxidant activity. With a molecular weight of 272.25 g/mol, naringenin possesses the ability to cross biological barriers, including the blood–brain barrier (BBB). Additionally, its moderate lipophilicity (log P ≈ 2.5–3.0) facilitates diffusion across cell membranes, enhancing bioavailability in the nervous system and other tissues [11].

The antioxidant activity of naringenin is associated with the scavenging of free radicals, particularly superoxide (O_2_^−^) and hydroxyl radicals (OH•), as well as the activation of the phosphatidylinositol 3-kinase (PI3K)/protein kinase B (Akt) and ERK signaling pathways, which in turn directly increases the activity of the endogenous enzymatic antioxidant system (superoxide dismutase—SOD, catalase—CAT, and glutathione peroxidase—GPx) [12,13,14]. Additionally, naringenin inhibits metal chelation and pro-oxidant enzymes such as nicotinamide adenine dinucleotide phosphate (NADPH), oxidase (NOX), xanthine oxidase (XDH), lipoxygenase (LOX), and cyclooxygenase (COX), providing cellular protection against oxidative stress, which is a key factor in the pathogenesis of diseases such as atherosclerosis, diabetes, and cancer. The anti-inflammatory properties of naringenin are mediated through the inhibition of nuclear factor kappa B (NF-κB) signaling, which regulates the expression of pro-inflammatory cytokines (e.g., tumor necrosis factor-α (TNF-α) and interleukin-6 (IL-6)) and enzymes such as COX-2, thereby mitigating inflammation and tissue damage associated with chronic inflammatory conditions [15,16,17].

Beyond its antioxidant and anti-inflammatory effects, naringenin exerts beneficial effects on lipid and carbohydrate metabolism. It enhances insulin sensitivity through the modulation of insulin receptor activity and glucose transporter (GLUT) function, suggesting potential applications in type 2 diabetes management [15,18,19]. Furthermore, naringenin reduces serum low-density lipoprotein (LDL) cholesterol and triglyceride levels while increasing high-density lipoprotein (HDL) cholesterol, contributing to cardiovascular protection [20,21]. Additionally, its hepatoprotective properties help safeguard liver cells from toxins and oxidative stress while supporting tissue regeneration [21].

### 2.2. Hesperidin

Hesperidin ((2*S*)-3′,5-dihydroxy-4′-methoxy-7-[α-l-rhamnopyranosyl-(1→6)-β-d-glucopyranosyloxy]flavan-4-one) is primarily found in citrus fruits such as oranges, lemons, and grapefruits. It is a glycoside derivative of hesperetin, in which the aglycone is conjugated with rutinose, a disaccharide composed of rhamnose and glucose. This structure confers a relatively high molecular weight (610.56 g/mol), water solubility, and polarity, affecting its bioavailability [22].

**Figure 2:**
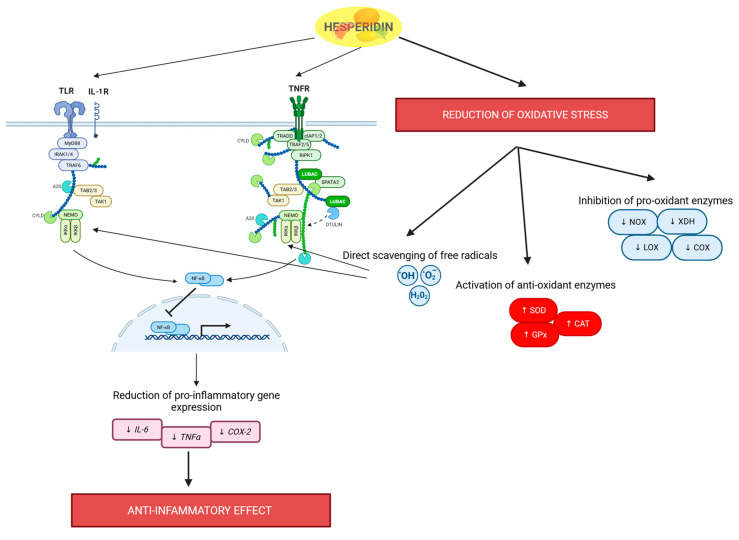
Mechanism of hesperidin action on the NF-κB signaling pathway. Hesperidin inhibits the activation of the NF-κB pathway by blocking surface receptors such as TLR4, IL-1R, and TNFR1, leading to the inhibition of the IKK complex and preventing the degradation of the inhibitor IκBα. As a result, the transcription factor NF-κB remains inactive in the cytoplasm, reducing the expression of pro-inflammatory genes (e.g., IL-6, TNF-α, IL-1β). Additionally, hesperidin neutralizes reactive oxygen species (ROS), reducing oxidative stress, which can also activate NF-κB. The overall effect of hesperidin is the reduction of inflammatory responses and oxidative stress. Abbreviations: A20—TNFAIP3 ubiquitin-editing enzyme; CAT—catalase; CIAP1/2—cellular inhibitor of apoptosis protein 1 and 2; COX—cyclooxygenase; COX-2—cyclooxygenase-2; CYLD—cylindromatosis lysine 63 deubiquitinase; GPx—glutathione peroxidase; H_2_O_2_—hydrogen peroxide; IKKα—IκB kinase alpha; IKKβ—IκB kinase beta; IL-1R—interleukin-1 receptor; IL-6—interleukin-6; IRAK1/4—interleukin-1 receptor-associated kinase 1 and 4; LOX—lipoxygenase; LUBAC—linear ubiquitin chain assembly complex; MyD88—myeloid differentiation primary response 88; NEMO—NF-κB essential modulator; NF-κB—nuclear factor kappa-light-chain-enhancer of activated B cells; NOX—NADPH oxidase; •O_2_^−^—superoxide anion radical; •OH—hydroxyl radical; RIPK1—receptor-interacting serine/threonine-protein kinase 1; SOD—superoxide dismutase; SPATA2—spermatogenesis-associated protein 2; TAB2/3—TAK1-binding proteins 2 and 3; TAK1—transforming growth factor-β-activated kinase 1; TLR4—toll-like receptor 4; TNF-α—tumor necrosis factor alpha; TNFR1—tumor necrosis factor receptor 1; TRADD—TNFR1-associated death domain protein; TRAF2/5—TNF receptor-associated factor 2 and 5; TRAF6—TNF receptor-associated factor 6; XDH—xanthine dehydrogenase. Created in BioRender. Bijak, M. (2025) https://BioRender.com/99h60kh, accessed on 15 May 2025 [25,29,30].

One of the principal mechanisms of action of hesperidin is its anti-inflammatory activity, mediated by inhibition of the NF-κB pathway, a key regulator of inflammatory processes [23,24]. Consequently, hesperidin reduces the production of pro-inflammatory cytokines such as IL-6 and TNF-α and suppresses COX-2 expression [25]. In addition to its anti-inflammatory properties, hesperidin demonstrates significant antioxidant activity, neutralizing reactive oxygen species (ROS), including superoxide anions (O_2_^−^), hydrogen peroxide (H_2_O_2_), and OH•. The presence of hydroxyl (-OH) groups in its structure allows electron donation, stabilizing free radicals, and interrupting oxidative chain reactions [26,27,28]. Moreover, hesperidin inhibits pro-oxidant enzymes (e.g., NOX, XDH, LOX, and COX) while enhancing the activity of antioxidant enzymes (SOD, CAT, GPx) (Figure 2) [29,30].

Hesperidin also exerts cardiovascular benefits by activating endothelial nitric oxide synthase (eNOS), increasing nitric oxide (NO) bioavailability, and promoting vasodilation [31]. Furthermore, it reduces the expression of adhesion molecules such as the intercellular adhesion molecule-1 (ICAM-1) and vascular cell adhesion molecule-1 (VCAM-1), which mediate leukocyte-endothelium interactions [32,33]. These effects contribute to improved vascular elasticity, reduced blood pressure, and a lower risk of atherosclerosis. Additionally, hesperidin lowers LDL cholesterol and triglycerides while increasing HDL cholesterol, further enhancing cardiovascular protection [34].

Beyond these effects, hesperidin exhibits antiviral activity, particularly against influenza and other respiratory viruses. This is attributed to its ability to inhibit viral replication and modulate protein activity, thus reducing overall infectivity [35,36,37]. Moreover, hesperidin possesses analgesic and antitumor properties, likely mediated through the modulation of inflammatory pathways and apoptosis regulation in cancer cells [38,39].

### 2.3. Flavanone Metabolism in the Gastrointestinal Tract

Dietary flavanones are primarily consumed in glycosylated forms. Upon ingestion, these compounds undergo enzymatic and microbial transformations in the gastrointestinal tract, leading to the hydrolysis of glycosidic bonds and the release of their biologically active aglycone forms. This conversion is essential for their absorption and subsequent therapeutic effects [40].

**Figure 3:**
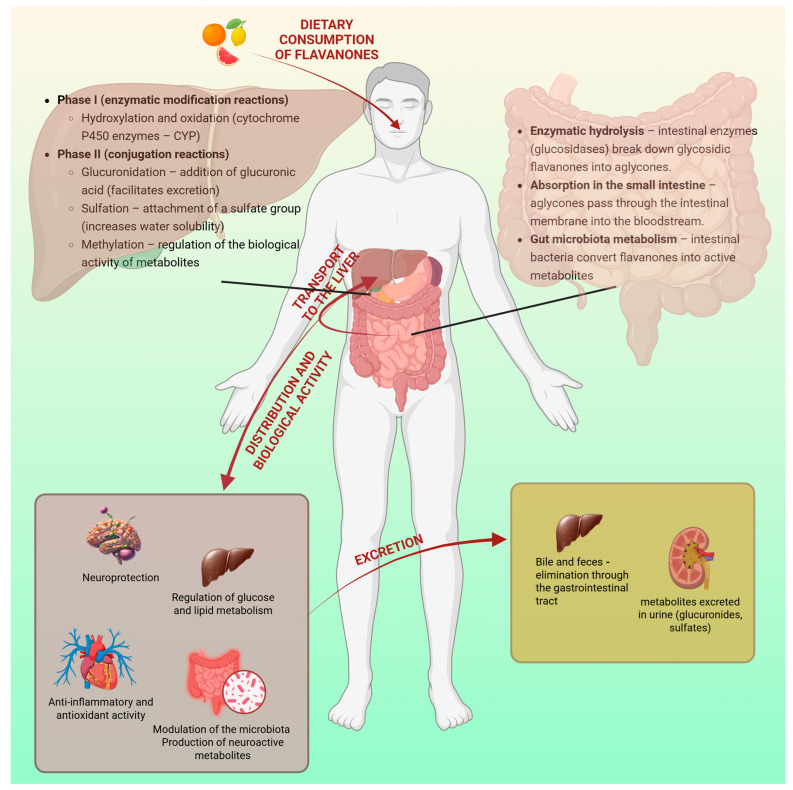
Flavanone metabolism and biological activity. Following dietary intake, flavanones undergo enzymatic hydrolysis, intestinal absorption, and microbial biotransformation. Hepatic phase I and II metabolism further modulates their bioavailability and activity. Flavanone metabolites contribute to neuroprotection, anti-inflammatory effects, and metabolic regulation, with elimination occurring via bile, feces, and renal excretion. Created in BioRender. Bijak, M. (2025) https://BioRender.com/z74d094, accessed on 15 May 2025 [41,42,43].

Glucosidases serve as the primary enzymes responsible for hydrolyzing the glycosidic bonds of flavanones, leading to the release of the biologically active aglycone. This transformation is essential for flavanones to exert their full range of physiological effects. Following this hydrolysis, glucuronidases and sulfatases further modify the liberated aglycones by conjugating them with glucuronic acid and sulfonic acid groups, respectively. These modifications enhance the hydrophilicity of flavanones, thereby improving their solubility, increasing bioavailability, and facilitating efficient absorption across the intestinal epithelium. Additionally, these metabolic modifications play a crucial role in the systemic distribution of flavanones, enabling their transport to the liver and other target tissues where they can exert therapeutic effects. Conjugation also enhances molecular stability, reducing susceptibility to rapid degradation and prolonging their biological activity within the body. Once aglycones are released from their glycosylated precursors, their bioavailability and biological potency significantly increase. Aglycones exhibit greater lipophilicity than their glycosidic counterparts, allowing them to more readily permeate cellular membranes, including the intestinal barrier. This enhanced membrane permeability facilitates their rapid absorption into the bloodstream, enabling their distribution to various organs such as the liver, brain, and heart, where they can mediate their pharmacological effects (Figure 3) [41,42,43].

Following absorption, the liberated aglycones undergo further biotransformation within the body, primarily in the liver. Flavanones can be subjected to metabolic modifications such as methylation, glucuronidation, and sulfation, which play crucial roles in their detoxification, conjugation, and subsequent excretion. These transformations enhance the compounds’ solubility and facilitate their elimination while preserving their pharmacological efficacy. Although these metabolic modifications primarily serve to regulate flavanone bioavailability and clearance, they may also influence their biological activity, potentially modulating their therapeutic effects before excretion [42,43].

## 3. Intestinal Microbiota and Flavanone Impact

The human microbiota, often called the “hidden organ”, is a complex community of microorganisms, including bacteria, fungi, archaea, and viruses, that inhabit various parts of the body such as the gut, skin, mouth, and lungs. The microbial population considered as a human microbiota contributes more than 150 times the genetic material found in the human genome and plays a fundamental role in maintaining health and preventing various disease entities. While the term “microbiota” and “microbiome” are frequently used interchangeably, microbiota specifically refers to the living microorganisms in a particular environment, while microbiome includes their collective genomes, structural components, metabolites, and interactions with the environment. Among various types of microbiota, the gut microbiota is the most crucial for human health [44,45].

The composition of microbial populations varies along different segments of the gastrointestinal tract, reflecting the distinct environmental conditions and functions of each region. Bacterial density increases progressively throughout the digestive system, starting at approximately 10^5^ colony-forming units per gram (cfu/g) in the jejunum, where genera such as *Bacteroides*, *Lactobacillus*, and *Streptococcus* are present, and rising to 10^8^ cfu/g in the ileum, which is primarily populated by *Bacteroides*, *Clostridium*, *Enterococcus*, *Lactobacillus*, *Veillonella*, and *Enterobacteriaceae* [46]. The highest microbial density and diversity are found in the large intestine, which hosts the most metabolically active bacterial communities. The total mass of gut microbiota is estimated to range from 1.5 to 2 kg, with the dominant bacterial phyla including *Firmicutes*, *Bacteroidetes*, *Proteobacteria*, and *Actinobacteria*. Research suggests that bacterial concentrations in the large intestine can reach 10^12^ cells per gram of content, with an estimated 800 to 900 species, including both bacteria and archaea. The microbial ecosystem of the large intestine is primarily composed of obligate anaerobes, such as *Bacteroides*, *Clostridium*, *Ruminococcus*, *Fusobacterium*, *Butyrivibrio*, *Peptostreptococcus*, *Eubacterium*, and *Bifidobacterium*, alongside aerobic and facultatively anaerobic bacteria, including Gram-negative rods from the *Enterobacteriaceae* family, Gram-positive rods from the *Lactobacillus* genus, and cocci from the *Enterococcus* and *Streptococcus* genera. In addition, small populations of fungi, mainly *Candida* spp., are present at concentrations ranging from 10^2^ to 10^4^ cells per gram of stool [45,46].

Notably, approximately 80% of the bacteria residing in the gut cannot be cultivated using standard microbiological techniques. However, about 30% of microbial species display relatively stable population sizes, forming a core microbiota that is consistently present across individuals. The remaining microbial populations are highly dynamic and subject to variation due to factors such as immune system function, genetic background, dietary habits, physical activity, and environmental influences [45,47]. Understanding these complex interactions is essential for elucidating the role of gut microbiota in maintaining human health and its potential involvement in disease development.

### Flavanones Change the Composition in Gut Microbiota

In a study conducted by Wu et al. [48], naringenin was administered to the Sprague–Dawley rats with induced polycystic ovary syndrome (PCOS) to investigate, inter alia, its impact on gut microbiota composition. The authors compared the microbial changes at the genus level in four groups: (1) the normal group, in which rats received only diluent carboxymethyl cellulose; (2) the Diane-35 group, where rats were given Diane-35 (0.2 mg/kg/day) and constituted as a positive control; (3) the naringenin group, which received naringenin (20 mg/kg/day); and (4) the PCOS-like group, in which rats were treated with Letrozole (1 mg/kg/day) dissolved in carboxymethyl cellulose.

The results showed a significant shift in the species composition of the gut microbiota after naringenin administration. Compared to the normal group, the abundance of genera such as *Helicobacter*, *Dorea*, *Lachnospira*, *Butyricimonas*, *Roseburia*, *Streptococcus*, *Parabacteroides*, *Phascolarctobacterium*, *Blauria*, *Butyricicoccus*, *Paraprevotella*, *Coprococcus*, *Bosea*, and *Coprobacillus* was elevated in the naringenin group. Conversely, genera such as *Akkermansia*, *Clostridium*, *Dehalobacterium*, *Pseudoxanthomonas*, *Bacillus*, *Desulfovibrio*, and *Fusobacterium* were diminished. Furthermore, compared to the PCOS-like group, naringenin treatment reduced the level of *Gemella*, *Prevotella*, *Fusobacterium*, and *Veillonella*, and increased the contents of *Blautia*, *Helicobacter*, *Ruminococcus*, *Lactobacillus*, *Coprococcus*, *Faecalibacterium*, *Parabacteroides*, *Streptococcus*, *Roseburia*, *Paraprevotella*, and *Butyricicoccus* [48].

In a study conducted by Liu et al. [49], naringenin was administered to naïve mice to evaluate its impact on gut microbiota composition. The authors analyzed operational taxonomic units (OTUs) based on 16S rRNA sequencing after four weeks of naringenin treatment. The Venn diagram revealed that the naringenin-treated group had nearly 2.5 times more OTUs than the naïve control group, indicating a substantial increase in microbial diversity. A taxonomic analysis showed that in the naringenin group, the most abundant bacterial class was Betaproteobacteria, with Burkholderiales, Methylophilales, and Turicibacterales being the dominant orders. In contrast, the control group exhibited a higher prevalence of the Gammaproteobacteria and Alphaproteobacteria class, with Enterobacteriales, RF32, Sphingomonadales, Fusobacteriales, Rhizobiales, and Rickettsiales as the most abundant bacterial orders. Additionally, the study examined the effects of naringenin on the gut microbiota of mice with experimental autoimmune encephalomyelitis (EAE). Mice fed with naringenin displayed a significantly higher number of bacterial species compared to the control group. In the naringenin-treated group, the most abundant taxa included Paraprevotellaceae, *Alistipes*, and Chlorobi, while levels of Bacteroidetes and *Akkermansia* were reduced. In contrast, the control group showed a predominance of Desulfovibrionaceae and Deltaproteobacteria [49].

In the Parkar et al. [50] study, *Staphylococcus aureus* was shown to be the most sensitive to naringenin and quercetin (62.5 µg/mL). *Escherichia coli*, *Salmonella typhimurium*, and *Lactobacillus rhamnosus*, were much less sensitive to naringenin, showing 2 times greater minimum inhibitory concentration (125 µg/mL) [50].

In a study performed by Duda-Chodak [51], naringin, naringenin, hesperetin, and hesperidin were examined for their effects on the growth of human intestinal bacteria, including *Bacteroides galacturonicus*, *Escherichia coli*, *Bifidobacterium catenulatum*, *Lactobacillus sp.*, *Enterococcus caccae*, and *Ruminococcus gauvreauii*. However, the study results did not show a beneficial effect of the tested flavanones on the intestinal microbiota, thus indicating that an increased number of polyphenolic compounds, such as flavanones, may inhibit the growth of beneficial bacteria in the gut. Research showed that naringenin partially or completely reduced the growth of the above-mentioned bacteria in a dose-dependent manner. A similar but not that spectacular effect was observed for hesperetin. Furthermore, the study confirmed that the glycosylated forms of flavanones (naringin and hesperidin) exhibited a significantly lower bioavailability [51]. In contrast, Firrman et al. showed that naringenin enhanced the growth of *Bifidobacterium catenulatum*, did not affect the growth of *Ruminococcus gauvreauii*, and strongly inhibited the growth of *Enterococcus caccae* [52]. The influence of hesperetin and naringenin was also shown in the Bae et al. study, in which the growth of Helicobacter pylori strains was inhibited by 57% and 34%, respectively [53].

In a study by Unno et al. [54], hesperetin and hesperidin were administered to Wistar rats to assess their impact on gut microbiota composition. The results showed that consumption of 0.5% hesperetin led to a significant decrease in *Clostridium* spp. Additionally, hesperetin administration caused a slight increase in the *Lactobacillales*, *Bifidobacterium*, and *Bacteroides*, while *Prevotella* levels decreased; however, these changes were not statistically significant. In addition to analyzing gut microbiota composition, the researchers demonstrated that hesperetin, unlike its glycoside hesperidin, influenced starch excretion in feces and SCFA pools in the cecal content. Moreover, hesperetin exhibited a stronger inhibitory effect on starch digestion compared to hesperidin, thus confirming that the attachment of a rutinose moiety to the aglycone may reduce its effectiveness [54].

A recent study demonstrated that dietary supplementation with orange juice (OJ) can modulate gut microbiota composition, influencing host metabolism and metabolic health. A significant increase in Actinobacteria (*p* = 0.003) was observed following the OJ-Diet, primarily driven by the higher abundance of Bifidobacteriaceae (*p* = 0.002), Atopobiaceae (*p* = 0.001), Coriobacteriaceae (*p* = 0.005), and Eggerthellaceae (*p* = 0.002) families. Conversely, the relative abundance of Bacteroidetes decreased, with a greater representation of specific families, including Bacteroidaceae, Barnesiellaceae, Muribaculaceae, Prevotellaceae, Rikenellaceae, and Tannerellaceae [55]. Furthermore, in the OJ-Diet, a reduction in the overall abundance of Firmicutes was observed; however, specific families within this phylum, including Lactobacillaceae, Leuconostocaceae, Clostridiaceae 1, Lachnospiraceae, Peptococcaceae, and Ruminococcaceae, exhibited a relative increase, suggesting a selective modulation of gut microbiota composition in response to flavanone-rich orange juice consumption. Interestingly, *Akkermansia muciniphila*, a microorganism inversely associated with metabolic disorders and considered as a potential beneficial next-generation probiotic, was significantly augmented in the OJ-Diet group compared to OJ-Free Diet group [55].

In a controlled clinical study with a temporal series intergroup design conducted by Lima et al. [56], 10 apparently healthy women (28.5 ± 8.4 years; BMI 24.1 ± 3.3 kg/m^2^) consumed commercial pasteurized orange juice daily for two months. Gut microbiota was assessed through quantitative cultures (*Lactobacillus*, *Bifidobacterium*, *Clostridium*, and total anaerobic bacteria population) and a DGGE analysis, alongside metabolic parameters such as pH, ammonium, and short-chain fatty acids. The results showed a significant increase in beneficial bacteria populations and improvements in metabolic markers, including reductions in LDL-cholesterol, glucose levels, and enhanced insulin sensitivity [56]. Duque et al. performed a study in which the Simulator of the Human Intestinal Microbial Ecosystem (SHIME^®^) was used [57]. SHIME^®^ is an advanced in vitro model that replicates the human gastrointestinal tract to study interactions between gut microbiota and various substances, such as food components, pharmaceuticals, and microbial interventions in controlled environmental conditions such as pH, temperature, or retention time. The SHIME^®^ consists of special double-jacketed vessels that represent parts of the GI tract such as the stomach, small intestine, ascending colon (AC), the transverse colon (TC), and the descending colon (DC) [57,58]. In the obtained results, fresh OJ treatment increased the growth of *Lactobacillus* and *Enterococcus* bacteria (1logCFU) in all three regions of the colon (*p* ≤ 0.05). Furthermore, the 1logCFU elevation was observed for *Bifidobacterium* in the TC and DC region (*p* ≤ 0.05) and for *Clostridium* in AC and TC (*p* ≤ 0.05). On the other hand, 1logFCU reduction (*p* ≤ 0.05) in the Enterobacteria population in the AC was observed [57]. The growth of Bifidobacterium and Lactobacillus in the colon positively impacts gut health by supporting immune system regulation and promoting the production of short-chain fatty acids (SCFAs). Additionally, these bacterial populations contribute to antimicrobial defense by producing bacteriocins, which help limit the presence of pathogenic microorganisms. An increased abundance of Clostridium may also have positive implications since certain species within the genus are associated with the production of SCFAs, which play a beneficial role in health [57,59]. All changes in gut microbiota composition in response to flavanone administration are summarized in Table 1.

## 4. The Gut–Brain Axis and Cognitive Health

The gut–brain axis (GBA) is a complex, bidirectional communication network linking the gastrointestinal tract to the CNS. This connection is essential for maintaining cognitive function and overall brain health. Moreover, the gut microbiota plays a pivotal role in modulating the GBA, highlighting the significance of GBA research as a promising area of therapeutic interest, particularly in the context of cognitive disorders and neurodegenerative disease. The intestinal microbiota consists of a vast and diverse community of bacteria that play a crucial role in various physiological processes. Maintaining an appropriate quantitative and qualitative microbial composition, known as eubiosis, is essential for sustaining systemic homeostasis, modulating immune function, regulating metabolism, and synthesizing numerous bioactive compounds [45]. Since certain bacterial species within the gut can exhibit pathogenic characteristics, beneficial microorganisms must predominate, as they contribute to essential intestinal processes that positively influence overall health [60].

### 4.1. Significance of GBA in Cognitive Health

The proper development of the gut microbiota plays a fundamental role in shaping nervous system function, with its foundation established as early as the prenatal period. Maternal health, diet, stress levels, and infections during pregnancy significantly influence the composition of the infant’s microbiota, and, consequently, neurodevelopment. Disruptions in this process have been associated with an increased risk of neurodevelopmental disorders, including autism, ADHD, and schizophrenia. The bidirectional communication between the gut microbiota and the CNS involves multiple mechanisms, including microbiota composition modulation, immune system interactions, vagus nerve signaling, tryptophan metabolism, intestinal hormonal responses, and bacterial metabolites [61].

### 4.2. GBA and Neurotransmitters

The gut microbiota plays a fundamental role in the synthesis, metabolism, and regulation of various neurotransmitters, including serotonin, dopamine, and gamma-aminobutyric acid (GABA), which are essential for mood regulation, cognitive function, and overall mental health. These neurotransmitters facilitate communication between neurons and contribute to emotional stability, memory, and learning processes [62].

Serotonin (5-hydroxytryptamine, 5-HT), in addition to its role as a neurotransmitter in the CNS, plays a crucial regulatory function in the gastrointestinal tract and other organ systems, influencing a range of physiological processes. Over 90% of serotonin in the body is synthesized in the intestines, primarily by enterochromaffin cells (EC), as well as by mucosal mast cells and enteric neurons. Specific bacterial strains, such as *Lactobacillus* and *Bifidobacterium*, have been shown to influence serotonin synthesis by modulating the availability of its precursor, tryptophan [63]. Intestinal serotonin interacts with 14 distinct 5-HT receptor subtypes located on enterocytes, enteric neurons, and immune cells [63,64,65]. Furthermore, circulating platelets sequester serotonin from the gastrointestinal tract, releasing it to regulate hemostasis and facilitate its distribution to various tissues. Intestinal serotonin plays a key role in modulating intestinal motor and secretory reflexes, platelet aggregation, immune responses, as well as in the regulation of bone development and heart function [66]. Despite significant advances in research, the precise mechanisms controlling serotonin metabolism in the gut remain unknown. Yano et al. demonstrated the critical role of the host microbiota in 5-HT biosynthesis, identifying specific fecal metabolites, such as SCFAs, particularly butyrate, which influence serotonin synthesis by modulating the function of EC [67]. Disruptions in the gut microbiota composition, often due to stress, dietary factors, infections, or antibiotic use, can lead to alterations in serotonin levels, potentially contributing to mood disorders such as depression and anxiety, as well as cognitive impairments.

Similarly, dopamine, a key neurotransmitter involved in cognitive functions, reward processing, and motor control, is closely linked to the GBA. Research indicates that the gut microbiota plays a crucial role in dopamine metabolism through bidirectional communication pathways, including the vagus nerve, immune signaling, and microbial metabolites [68]. The vagus nerve, a major component of the parasympathetic nervous system, serves as a critical conduit for GBA. Studies in animal models have demonstrated that gut microbiota can modulate central dopaminergic pathways via vagal stimulation, thereby influencing behavioral responses [69,70]. Immune signaling represents another mechanism through which the gut microbiota influences dopamine metabolism. Gut microbes regulate host immune responses by modulating the production of pro- and anti-inflammatory cytokines. Dysbiosis, or microbial imbalance, can contribute to chronic inflammation, which in turn affects CNS function, including dopaminergic pathways. Experimental studies have shown that inflammation can alter dopamine synthesis, release, and metabolism, potentially contributing to neurological disorders such as depression and Parkinson’s disease [71]. Additionally, microbial metabolites, particularly SCFAs such as butyrate, propionate, and acetate, play a significant role in dopaminergic modulation. Studies in animal models have demonstrated that SCFAs can regulate the expression of genes involved in dopamine synthesis and metabolism in the brain, thereby affecting reward-related behaviors and motivation. In a study conducted by van de Wouw et al. it was demonstrated that the administration of SCFAs to mice increased the expression of the dopamine D1a receptor (DRD1a) in the striatum, regardless of psychosocial stress. This finding suggests that SCFAs may directly influence dopaminergic pathways in the brain [72]. Furthermore, Ostendorf et al. reported that SCFAs can promote the synthesis of tyrosine hydroxylase (TH), a key enzyme in dopamine biosynthesis, leading to increased dopamine levels in the brain. This discovery highlights the potential role of SCFAs in modulating dopaminergic activity by influencing key enzymes involved in dopamine production [73].

Gamma-aminobutyric acid (GABA), the primary inhibitory neurotransmitter in the CNS, plays a crucial role in reducing neuronal excitability and promoting relaxation. Several gut-resident bacteria, notably strains of *Lactobacillus* and *Bifidobacterium*, have been identified as significant producers of GABA. In the study conducted by Yunes et al. 135 human-derived bacterial strains were screened for their ability to produce gamma-aminobutyric acid (GABA). The results demonstrated that 58 of these strains were capable of GABA synthesis, with the *Bifidobacterium* species exhibiting particularly high production levels, reaching up to 6 g/L [74]. The synthesis of GABA in these bacteria was primarily facilitated by the enzyme glutamate decarboxylase (GAD), which converts glutamate to GABA. The presence of GAD-encoding genes in these strains underscores their potential to influence host neurophysiology through GABA production [75]. The synthesis of GABA in these bacteria is primarily facilitated by the enzyme glutamate decarboxylase (GAD), which converts glutamate to GABA, thus highlighting the potential influence of the indicated strains in the modulation of host neurophysiology [76]. The GBA serves as a bidirectional communication pathway, allowing gut-derived GABA to influence CNS function. Studies have shown that modulation of the gut microbiota can impact brain GABA levels, thereby affecting behavior and emotional regulation [77].

### 4.3. Immune System

The gut microbiota plays a crucial role in modulating the immune system, influencing systemic inflammation and immune responses that can impact brain health. Chronic low-grade inflammation is characteristic of neurodegenerative diseases such as Alzheimer’s and Parkinson’s disease. Through its effects on inflammatory pathways, the microbiota can influence neuroinflammation, and, consequently, cognitive decline.

One of the primary mechanisms by which the gut microbiota influences the immune system is through the production of SCFAs, such as acetate, propionate, and butyrate. These metabolites, produced by the fermentation of dietary fiber by gut bacteria, exhibit anti-inflammatory properties. SCFAs can modulate immune responses by inhibiting the production of pro-inflammatory cytokines and promoting the differentiation of regulatory T cells, which is crucial for maintaining immune homeostasis. A study by Liu et al. demonstrated that SCFAs significantly attenuated behavioral impairment and neuronal degeneration and decreased the levels of IL-1β and IL-6 in the brains of mice with sepsis-associated encephalopathy [78].

Braniste et al. demonstrated that the gut microbiota influenced (BBB) permeability in mice. They found that germ-free mice exhibited increased BBB permeability compared to pathogen-free mice with a normal gut flora. This increased permeability was associated with the reduced expression of tight junction proteins, such as occludin and claudin-5, which are known to regulate barrier function in endothelial tissues. Furthermore, the exposure of germ-free adult mice to a pathogen-free gut microbiota decreased BBB permeability and upregulated the expression of these tight junction proteins [79].

Metabolites produced by the gut microbiota are increasingly recognized for their profound effects on brain function and cognitive health. Butyrate acts as a histone deacetylase inhibitor, which facilitates the acetylation of histones and leads to the activation of genes involved in neurogenesis and synaptic plasticity [80,81].

Recent studies have underscored the significant role of SCFAs in modulating cognitive functions through the regulation of the brain-derived neurotrophic factor (BDNF), a critical protein involved in neuronal survival, synaptic plasticity, and memory formation. A study conducted by Li et al. demonstrated that sodium butyrate effectively alleviated lead-induced neuroinflammation and cognitive impairments by activating the ACSS2/H3K9ac/BDNF pathway. This finding suggests that SCFAs may exert protective effects on brain function through the attenuation of inflammation and the promotion of neurotrophic signaling, thereby enhancing cognitive performance [82]. Collectively, these findings suggest that SCFAs, via their modulation of BDNF expression, play a pivotal role in supporting cognitive functions, particularly those related to memory and learning. By promoting neurogenesis and protecting neuronal cells from oxidative stress and inflammation, SCFAs hold promise as potential therapeutic agents for cognitive decline, including in neurodegenerative diseases such as Alzheimer’s and Parkinson’s disease.

## 5. Potential Applications for Cognitive Health

**Figure 4:**
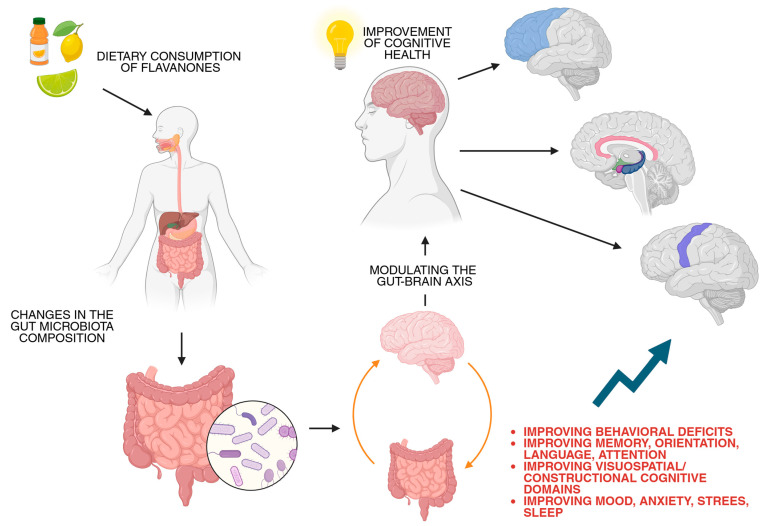
Summary of the contribution of gut microbiota modulation induced by flavanone consumption to cognitive function improvement. Created in BioRender. Bijak, M. (2025) https://BioRender.com/s2fqc7t, accessed on 15 May 2025 [85,86,87,90,91,92,95,96,97,98].

Recent studies highlight the potential for the modulation of cognitive functions and mitigation of neurodegenerative processes by gut microbiota. These studies examined how the therapeutic potential of probiotics, prebiotics, dietary modifications, and microbiota transplantation impacts the gut–brain–axis connection (GBA) in supporting cognitive health (Figure 4) [83].

Probiotics, live microorganisms that confer health benefits when administered in adequate amounts, have been widely studied for their role in cognitive function. Preclinical studies have demonstrated that probiotic supplementation can influence neurotransmitter production, reduce neuroinflammation, and promote hippocampal neurogenesis. Supplementation with *Lactobacillus helveticus* NS8 in a rat model of chronic restraint stress improved anxiety, depression, and cognitive function, showing comparable or superior effects to the selective serotonin reuptake inhibitor (SSRI) citalopram. This treatment also reduced plasma corticosterone and adrenocorticotropic hormone levels, increased IL-10, and restored serotonin and norepinephrine levels in the hippocampus, along with increased BDNF expression [84]. Similarly, the administration of *Bifidobacterium breve* Bif11 in LPS-induced depression-like mice prevented behavioral deficits and reduced inflammatory cytokines, such as TNF-α and IL-6. Additionally, it restored BDNF levels and improved gut permeability and SCFA profiles [85].

Clinical studies provide further support for the beneficial effects of probiotics on cognitive function. In a randomized controlled trial (RCT), Akbari et al. [86] demonstrated that a 12-week supplementation with a probiotic mixture containing *Lactobacillus acidophilus*, *Lactobacillus casei*, *Bifidobacterium bifidum*, and *Lactobacillus fermentum* improved Mini-Mental State Examination (MMSE) scores in patients with Alzheimer’s disease. In this randomized, double-blind, controlled trial, 60 patients were divided into two groups (n = 30), receiving either probiotic-enriched milk (intervention) or regular milk (comparator). This cognitive improvement was accompanied by reduced oxidative stress and improved metabolic parameters, including insulin sensitivity and decreased levels of inflammatory markers [86]. In turn, Mo et al. [87] conducted a meta-analysis to assess the impact of probiotics on cognitive function in individuals with mild cognitive impairment (MCI) and Alzheimer’s disease. Twelve randomized controlled trials (RCTs) involving 852 patients were included. The analysis revealed significant improvements in global cognitive function, recall/delayed memory, attention, and the visuospatial/constructional cognitive domains following probiotic supplementation. These findings suggest that probiotics may be an effective intervention for enhancing cognitive performance in individuals with MCI and AD [87].

Prebiotics, non-digestible dietary fibers that selectively stimulate the growth of beneficial gut bacteria, have demonstrated neuroprotective effects through various mechanisms. Prebiotics such as fructooligosaccharides (FOS) and galactooligosaccharides (GOS) enhance SCFAs production [88]. Furthermore, De Paiva et al. investigated the effects of FOS and GOS on neuroinflammation and cognitive function in mice fed a high-fat diet (HFD). The study showed that FOS and GOS supplementation reduced serum IL-1β levels and decreased neuroinflammatory markers such as TNF-α, COX-2, and Iba-1. The prebiotics also promoted synaptic plasticity by increasing markers like BDNF and CREB-p, and improved spatial learning and memory. Furthermore, FOS and GOS modulated the gut microbiota, restoring the balance of Bacteroidetes, reducing intestinal inflammation, and improving gut permeability [89].

Clinical trials support the potential cognitive benefits of prebiotic supplementation. In the 12-week PROMOTe trial, a double-blind, placebo-controlled RCT, cognitive outcomes were evaluated in older adults using the CANTAB cognitive battery. Participants were healthy twins aged 60 years and above, recruited from the TwinsUK cohort, with a low baseline protein intake but no specific cognitive complaints. They were randomized into two groups: one received prebiotic supplementation with protein, and the other received a placebo with protein. While the prebiotic intervention did not affect muscle strength, it significantly improved cognitive performance (coefficient 0.482; *p* = 0.014) compared to the placebo group [90]. Conversely, another trial reported that prebiotic supplementation did not enhance reading or cognitive performance in primary school children. This proof-of-concept study investigated the effects of prebiotic supplementation on cognitive function in children aged 7 to 9 with low reading scores. The study was conducted over a 3-month period, with participants receiving either a prebiotic supplement or a placebo. In addition to cognition, the study assessed secondary outcomes including sleep, behavior, mood, anxiety, and cortisol levels. However, the results indicated that the prebiotic supplementation did not significantly influence any of the measured outcomes [91]. Additionally, a study on older adults with moderate psychological distress indicated that a high-prebiotic diet improved mood, anxiety, stress, and sleep, suggesting potential cognitive benefits. The study “Gut Feelings” was an 8-week, 2 × 2 factorial randomized controlled trial involving 119 adults with moderate psychological distress and low prebiotic intake. The primary outcome was the assessment of total mood disturbance (TMD) using the Profile of Mood States Short Form, with secondary outcomes including anxiety, depression, stress, sleep, and wellbeing measures. The results showed that the high-prebiotic diet significantly reduced TMD compared to the placebo (Cohen’s d = −0.60, *p* = 0.039), with improvements in anxiety, stress, and sleep. However, no significant improvements were observed with probiotic supplementation or the synbiotic combination, and cognitive performance was not directly assessed [92]. Postbiotics, bioactive compounds derived from bacterial metabolism, have emerged as potential modulators of brain function. These metabolites include SCFAs, tryptophan derivatives, and anti-inflammatory peptides. Wu et al. investigated the neuroprotective effects of postbiotics derived from *Lactobacillus plantarum* in a *Salmonella* infection model. The study found that both heat-killed bacteria and their metabolites reduced neuroinflammation and cognitive impairments by lowering pro-inflammatory cytokines (IL-1β, IL-6) and increasing anti-inflammatory cytokines (IL-4, IL-10). The postbiotics also improved behavioral outcomes, modulated key neuroactive molecules, and favorably altered gut microbiota composition. These findings suggest that LP-derived postbiotics could offer a therapeutic approach for preventing brain dysfunctions associated with infection by targeting the GBA [93]. In addition to the neuroprotective effects of Lactobacillus plantarum-derived postbiotics, a study on heat-treated *Bifidobacterium longum* CECT 7347 (HT-ES1) suggests that postbiotics may also influence cognitive function by modulating gut health. HT-ES1 supplementation increased the abundance of Faecalibacterium and Anaerobutyricum, bacteria associated with butyrate production, a SCFA with known neuroprotective properties. While the primary focus was gastrointestinal health, these findings indicate that postbiotics may reduce neuroinflammation and support brain health through gut microbiota modulation, further supporting the potential role of postbiotics in enhancing cognitive function via the GBA [94].

Recent studies investigating fecal microbiota transplantation (FMT) have provided compelling evidence of its potential to modulate cognitive function via the GBA. In preclinical animal models, FMT has been shown to improve cognitive performance and alter neuroinflammatory processes. Jiang et al. [95] examined the effects of FMT on cognitive function in a 5× FAD mouse model of Alzheimer’s disease. After antibiotic treatment to deplete the gut microbiota, these mice received FMT either weekly or every other day. While weekly FMT did not produce significant improvements, administering FMT more frequently (every other day) successfully alleviated memory deficits, reduced amyloid β (Aβ) pathology, and decreased neuroinflammation. This suggests that the efficacy of FMT is time- and dose-dependent. The results highlight that restoring gut microbial diversity through FMT may play a role in mitigating cognitive decline, potentially by regulating molecular pathways involved in AD pathology, such as the Toll-like receptor 4/NF-κB signaling pathway [95]. Additionally, Cerna et al. [96] explored the effects of FMT from young, trained donors on cognitive function in aged mice. Their study demonstrated that FMT significantly improved cognitive performance, particularly in recognition and spatial memory, and enhanced long-term potentiation (LTP) in the hippocampus. These cognitive improvements were associated with reduced neuroinflammation and increased synaptic plasticity. The study also observed changes in gut microbiota composition, with higher levels of beneficial bacteria, such as *Akkermansia* and *Prevotellaceae*. Furthermore, levels of SCFAs, including butyrate, were elevated in the FMT-treated mice, which are known to exert neuroprotective effects [96].

In clinical settings, FMT has also shown promise as a potential therapeutic intervention for cognitive impairment. Park et al. [97] investigated the effects of FMT in 10 patients (age range, 63–90 years; 80% female) with dementia and severe Clostridioides difficile infection (CDI), who received FMT via colonoscopy, and compared them with a control group of 10 patients (age range, 62–91 years; 80% female) receiving antibiotics. Cognitive function was assessed using the Mini-Mental State Examination (MMSE) and Clinical Dementia Rating Scale Sum of Boxes (CDR-SB) at baseline and 1 month after treatment, with follow-up lasting 1 month. The results revealed significant cognitive improvements in the FMT group, with improvements in both clinical symptoms and cognitive function compared to the control group, suggesting that altering the gut microbiome can positively influence brain health in dementia patients. Additionally, changes in gut microbiota composition, such as an enrichment in *Proteobacteria* and *Bacteroidetes*, were also noted, further linking gut microbiota modulation with cognitive benefits. This study provided clinical evidence supporting FMT as an effective strategy to improve cognitive function in dementia patients, highlighting the gut–brain connection [97].

Chen et al. [98] conducted a pilot study evaluating the safety and efficacy of FMT in patients with mild cognitive impairment (MCI). In this study, patients who underwent FMT exhibited improvements or maintenance of cognitive function, especially in those with mild MCI, as measured by the Montreal Cognitive Assessment-B (MoCA-B) and Alzheimer’s Disease Assessment Scale-Cognitive (ADAS-Cog). FMT was also shown to alter the structure of gut microbiota and induce significant changes in serum metabolites. Notably, metabolites related to bile acid and choline metabolism were found to be altered, indicating a potential link between gut microbiota composition and cognitive health [98]. Collectively, these studies contribute to the expanding evidence base supporting FMT as a promising therapeutic approach for enhancing cognitive function. Whether applied in animal models of Alzheimer’s disease, clinical trials involving dementia patients, or interventions aimed at age-related cognitive decline, FMT shows significant potential in modulating gut microbiota, reducing inflammation, and ultimately improving cognitive health.

## 6. Limitations and Future Perspective

Despite their promising therapeutic potential, the clinical application of flavanones faces several challenges. One of the few limitations is their low bioavailability. After ingestion, flavanones undergo extensive metabolism in the liver and gut, rapidly forming metabolites with potentially altered biological activity. However, this limitation points to a crucial future perspective for studies focusing on flavanones. One of the most promising directions is the development of novel flavanone-based formulations with enhanced bioavailability. Advances in nanotechnology, such as nanoencapsulation and liposomal delivery systems, could improve their stability, absorption, and targeted delivery [99].

Another limitation is the varying content of flavanones in different citrus fruits and food products. Environmental factors, agricultural practices, and processing methods impact flavanone concentration, thus hindering the standardization process. Developing standardized extracts with consistent flavanone levels is essential for ensuring reproducible therapeutic effects [6].

Additionally, while flavanones have demonstrated safety in dietary consumption, their long-term effects at pharmacological doses remain unclear. Potential interactions with drugs, particularly those affecting the central nervous system, require further investigation. Future studies should examine possible adverse effects, including hepatotoxicity, nephrotoxicity, and gastrointestinal disturbances, especially in elderly populations and among individuals with pre-existing conditions and various disease entities. This limitation indicates the necessity for performing a large-scale, long-term clinical trial to validate flavanones’ effectiveness in humans. While preclinical models have demonstrated their neuroprotective effects, human studies remain limited. Future clinical trials should focus on determining optimal dosages, treatment durations, and potential side effects.

Another crucial aspect is elucidating the molecular mechanisms underlying flavanones’ neuroprotective effects. Understanding these pathways may enable the development of personalized nutrition and pharmacological interventions targeting the gut–brain axis.

## 7. Conclusions

The current available literature indicates a broad range of positive effects associated with the use of flavanones, emphasizing their antioxidant, anti-inflammatory, and neuroprotective effects. In this review, we focused on the ability of flavanones to modulate gut microbiota composition and the influence of specific microbial taxa on cognitive health. According to the data, flavanones affect the composition of gut microbiota, and their intake positively influences the growth of bacteria that support cognitive health. Therefore, it can be suggested that flavanones may indirectly contribute to the improvement of cognitive functions; however, confirmation of this hypothesis requires detailed laboratory studies and/or clinical trials.

## Figures and Tables

**Table 1:** The summary of the flavanones’ impact on changes in gut microbiota.

Study	Flavanone	Species	Studied Groups	Increased/Decreased After Flavanone Consumption	Reference
Wu et al.	Naringenin	*Helicobacter*, *Dorea*, *Lachnospira*, *Butyricimonas*, *Roseburia*, *Streptococcus*, *Parabacteroides*, *Phascolarctobacterium*, *Blauria*, *Butyricicoccus*, *Paraprevotella*, *Coprococcus*, *Bosea*, *Coprobacillus*	Sprague–Dawley rats	Increased	[48]
		*Akkermansia*, *Clostridium*, *Dehalobacterium*, *Pseudoxanthomonas*, *Bacillus*, *Desulfovibrio*, *Fusobacterium*		Decreased	[48]
		*Blautia*, *Helicobacter*, *Ruminococcus*, *Lactobacillus*, *Coprococcus*, *Faecalibacterium*, *Parabacteroides*, *Streptococcus*, *Roseburia*, *Paraprevotella*, *Butyricicoccus*	Sprague–Dawley rats with induced PCOS	Increased	[48]
		*Gemella*, *Prevotella*, *Fusobacterium*, *Veillonella*		Decreased	[48]
Liu et al.	Naringenin	Burkholderiales, Methylophilales, Turicibacterales	Naïve mice fed with naringenin	Increased	[49]
		Enterobacteriales, RF32, Sphingomonadales, Fusobacteriales, Rhizobiales, Rickettsiales	Naïve mice		[49]
		Paraprevotellaceae, *Alistipes*, Chlorobi	EAE mice	Increased	[49]
		Bacteroidetes, *Akkermansia*		Decreased	[49]
Parkar et al.		*Staphylococcus aureus*, *Escherichia coli*, *Salmonella typhimurium*, *Lactobacillus rhamnosus*	In vitro study	Decreased	[50]
Duda-Chodak et al.	Naringenin	*Bacteroides galacturonicus*, *Escherichia coli*, *Bifidobacterium catenulatum*, *Lactobacillus* spp., *Enterococcus caccae*, *Ruminococcus gauvreauii*	In vitro study	Decreased	[51]
	Hesperetin	*Bacteroides galacturonicus*, *Escherichia coli*, *Bifidobacterium catenulatum*, *Enterococcus caccae*, *Ruminococcus gauvreauii*		Decreased	[51]
		*Lactobacillus* spp.		Increased	[51]
Firrman et al.	Naringenin	*Bifidobacterium catenulatum*	In vitro study	Increased	[52]
		*Enterococcus caccae*		Decreased	[52]
Bae et al.	Naringenin and hesperitin	*Helicobacter pylori*	In vitro study	Decreased	[53]
Unno et al.	Hesperetin	*Clostridium* spp.	Wistar rats	Decreased	[54]
		*Lactobacilliales*, *Bifidobacterium*, *Bacteroides*		Increased	[54]
Fidélix et al.	Orange Juice	Actinobacteria, Bifidobacteriaceaem Atopobiaceae, Coriobacteriaceae, Eggerthellaceae, Lactobacilliaceae, Leuconostocaceae, Clostridiaceae 1, Lachnospiraceae, Peptococcaceae, Ruminococcaceae, *Akkermansia munciphila*	Women with the Orange Juice Diet	Increased	[55]
		Bacteroidaceae, Barnesiellaceae, Muribaculaceae, Prevotellaceae, Rikenellaceae, Tannerellaceae		Decreased	[55]
Lima et al.	Orange Juice	For *Lactobacillus*, *Bifidobacterium*, *Clostridium*, total anaerobic bacteria population	Women with the Orange Juice Diet	Increased	[56]
Duque et al.	Orange Juice	Lactobacillus Enterococcus, Bifidobacetrium, Clostridium	SHIME^®^ study	Increased	[57]
		Enterobacteria population in ascending colon		Decreased	[57]

Abbreviations: EAE—experimental autoimmune encephalomyelitis; PCOS—polycystic ovary syndrome; SHIME^®^—simulator of the human intestinal microbial ecosystem.

## Data Availability

No new data were created or analyzed in this study.
